# Does attachment get under our skin? Midlife attachment style predicts mild cognitive impairment and cumulative deficit frailty in old age

**DOI:** 10.1002/alz.091580

**Published:** 2025-01-09

**Authors:** Erik Buchholz, Carol E. Franz, Vietnam Era Twin Study of Aging (VETSA) group

**Affiliations:** ^1^ University of California, San Diego, La Jolla, CA USA; ^2^ University of Arkansas at Little Rock, Little Rock, AR USA

## Abstract

**Background:**

Poor social relations of older adults have been linked to cognitive decline, dementia risk, morbidity and mortality. However, this history of poor social relations may have started earlier in life and may be related to personality traits. Here we investigate how attachment style, a personality trait, can function as a protective factor for Alzheimer’s Disease and overall health. Specifically, we evaluated whether attachment style at midlife predicted two types of frailty in early old age: cognitive frailty and cumulative deficit frailty. Furthermore, to better understand these pathways, we have investigated several mediators: perceived stress, smoking, alcohol consumption, doctor visits, and social isolation.

**Method:**

Participants were 1275 community‐dwelling men from the Vietnam Era Twin Study of Aging (VETSA) at average ages 56, 62, and 68. Attachment style was assessed at age 56, health mediators at age 62. Frailty at age 68 included cognitive frailty (operationalized as mild cognitive impairment; MCI) and cumulative deficit frailty, a frailty index (FI) comprising 37 items assessing multiple physiological systems. Structural Equation Modeling (SEM) assessed mediators between attachment style and FI/MCI.

**Result:**

Age 56 attachment avoidance predicted age 68 MCI (β = 0.132); attachment anxiety predicted age 68 FI (β = 0.179). In mediation models, age 62 social isolation mediated associations between attachment avoidance (β = 0.169) and FI (β = 0.192). Attachment avoidance directly predicted MCI (β = 0.134). Age 62 perceived stress, doctor visits, and smoking acted as mediators between attachment anxiety (βs = 0.400, 0.069, 0.088, respectively) and FI (β = 0.284, 0.278, 0.134, respectively). Perceived stress mediated associations between attachment anxiety (β = 0.400) and MCI (β = 0.230). There was no direct effect of attachment anxiety on MCI.

**Conclusion:**

Personality traits involving viewing others as trustworthy and dependable may function as protective factors against frailty in old age. Attachment avoidance and anxiety influence frailty through different pathways, such as social isolation compared to perceived stress and smoking. Thus, to mitigate risk for either cognitive or cumulative deficit frailty, clinicians may wish to evaluate personality characteristics related to the quality of interpersonal relationships (e.g., attachment style) in addition to health factors.

